# Incidence of severe maternal outcomes following armed conflict in East Gojjam zone, Amhara region, Ethiopia: using the sub-Saharan Africa maternal near-miss criteria

**DOI:** 10.3389/fpubh.2024.1456841

**Published:** 2025-01-08

**Authors:** Tirusew Nigussie Kebede, Kidist Ayalew Abebe, Ambachew Getahun Malede, Abinet Sisay, Ayenew Yirdie, Worku Taye, Tebabere Moltot Kitaw, Bezawit Melak Fente, Mesfin Tadese, Tesfanesh Lemma Demisse, Mulualem Silesh, Solomon Hailemeskel Beshah, Getaneh Dejen Tiche, Michael Amera Tizazu, Moges Sisay Chekole, Birhan Tsegaw Taye

**Affiliations:** ^1^Department of Midwifery, Asrat Woldeyes Health Science Campus, Debre Berhan University, Debre Berhan, Ethiopia; ^2^Department of Midwifery, Debre Markos Comprehensive Specialized Hospital, Debre Markos, Ethiopia; ^3^Department of Obstetrics and Gynecology, Debre Markos Comprehensive Specialized Hospital, Debre Markos, Ethiopia; ^4^Department of General Midwifery, School of Midwifery, Collage of Medicine and Health Science, University of Gondar, Gondar, Ethiopia; ^5^Department of Obstetrics and Gynecology, Asrat Woldeyes Health Science Campus, Debre Berhan University, Debre Berhan, Ethiopia; ^6^Department of Public Health, Asrat Woldeyes Health Science Campus, Debre Berhan University, Debre Berhan, Ethiopia

**Keywords:** severe maternal outcome, uterine rupture, sub-Saharan Africa, life-threatening condition, hypertensive disorders of pregnancy, obstetrical hemorrhage

## Abstract

**Background:**

Severe maternal outcome (SMO) encompasses women who survive life-threatening conditions either by chance or due to treatment quality, or who die. This concept assumes that severe maternal morbidity predicts mortality risk, enabling the analysis of risk factors for life-threatening outcomes and improving our understanding on the causes of maternal death. This study aims to determine the incidence of SMO and its leading causes in East Gojjam during a period of regional conflict.

**Methods:**

A prospective follow-up study was conducted at Debre Markos Comprehensive Specialized Hospital in East Gojjam from July 1, 2023, to February 30, 2024. The study included 367 women admitted with potentially life-threatening conditions, including 8 maternal deaths, using sub-Saharan Africa (SSA) and WHO Maternal Near-Miss (MNM) criteria. Data were entered into Epi Data v.4.6 and analyzed using SPSS v.27. The WHO MNM approach assessed SMO indicators and maternal health care quality were utilized.

**Results:**

During the eight-month period, there were 3,167 live births, 359 potentially life-threatening conditions (PLTC), and 188 SMO cases (180 MNM and 8 maternal deaths). The SMO ratio was 59.4 per 1,000 live births (95% CI: 51, 68 per 1,000 live births). The MNM to mortality ratio, mortality index, and maternal mortality ratio were 22.5:1, 4.2%, and 252.6 per 100,000 live births, respectively. Over 80% of women with SMO showed evidence of organ dysfunction upon arrival or within 12 h of hospitalization. The leading causes of SMO were hypertensive disorders of pregnancy (HDP) and obstetric hemorrhage, including uterine rupture, with uterine rupture contributing to half of the maternal deaths.

**Conclusion:**

This study found that the incidence of SMO was comparable to that reported in most other studies. HDP was the primary cause of SMO, followed by obstetrical hemorrhage, consistent with other studies in Ethiopia. Uterine rupture was identified as the leading cause of maternal death. As this study was conducted in a single institution and in the period of severe armed conflict, it may not fully capture the range of maternal health issues across populations with varying healthcare access and socio-economic backgrounds. Caution should be exercised when generalizing these findings to the wider population.

## Introduction

Despite a 34% decrease in the maternal mortality ratio (MMR) from 2000 to 2020, 287,000 women died during or after pregnancy and childbirth in 2020. Nearly 95% of these deaths occurred in low and lower middle-income countries, and most of this death are preventable (1). The progress toward reducing maternal mortality is a critical target within the Millennium Development Goals (MDGs) and now integral to the Sustainable Development Goals (SDGs), has been uninspiring in many nations with elevated maternal mortality rates. Conversely, progress has either stalled or remains alarmingly high in numerous sub-Saharan African (SSA) countries (2, 3). Ethiopia has made significant progress in reducing its MMR, which fell from 871 per 100,000 live births in 2000 to 412 per 100,000 live births in 2016 (4). According to the United Nations (UN) Interagency MMR estimate for 2020, the figure further decreased to 267 per 100,000 live births. While this represents considerable improvement, it remains well above the SDG target of 70 per 100,000 live births (5).

Maternal mortality represents just a fraction of the total burden within the spectrum of maternal health issues (6). In addition to MMR, evaluating cases where women survived life-threatening complications during pregnancy, childbirth, or postpartum (referred to as maternal near miss or severe acute maternal morbidity) is gaining recognition as a valuable method for assessing the quality of obstetric care (7). This approach operates under the assumption that sever maternal morbidity predicts mortality risk, allows for analysis of risk factors for life-threatening outcomes, which may improve our understanding of the antecedents of maternal death (8). The vast majority of women experience life-threatening complications, often resulting in long-term disabilities even if they survive (9). Annually, a minimum of 40 million women faces the prospect of enduring a long-lasting health complication resulting from pregnancy and childbirth (10, 11). Severe maternal outcomes are maternal near miss cases (a woman who nearly died but survived a complication that occurred during pregnancy, childbirth or within 42 days of termination of pregnancy) and maternal death (12).

SMO encompasses a broad range of clinical conditions, including illnesses that pose a threat to a woman’s life throughout pregnancy, childbirth, and postpartum. This phenomenon is particularly prevalent in low-income countries, with rates reaching up to 32.7 to 1,088 per 1,000 live births (13–18). In Ethiopia, the incidence of SMO ranges widely from 37.5 to 84 cases per 1,000 live births, this variance underscores the diverse healthcare challenges and disparities faced across different regions within the country (19–21). Uterine rupture and obstructed labor, alongside hypertensive disorders, obstetric hemorrhage, severe anemia, and geographical location have been pinpointed as primary factors contributing to SMO. The particular concerns are the high case fatality rates associated with eclampsia and obstetric hemorrhage (13, 15, 17, 20, 21).

Severe armed conflict has contributed up to 0.3 million maternal deaths in conflict-affected areas (22). Maternal health can be significantly impacted by armed conflicts through the disruption of essential reproductive health services, including limited access to obstetric care and contraceptive methods (23). The long-term impacts of war are evident, with increases in maternal mortality observed for up to 7 years after the conflict (22). In Ethiopia, during the armed conflict in the northern region, maternal health services were severely affected. Antenatal care (ANC) coverage decreased by 62.3%, institutional deliveries dropped by 72.7%, and postnatal care (PNC) declined by 71.8% (24). Additionally, MMR in the region rose to 840 per 100,000 live births (25). Therefore, severe armed conflicts significantly contribute to SMOs in affected areas through various dimensions.

While there is some existing evidence on the burden of maternal near miss in Ethiopia, it remains insufficient, particularly in addressing the designated study area and the incidence of maternal mortality. This study endeavors to bridge this gap by using the SSA MNM criteria. It will specifically investigate the period following the onset of armed conflict in the Amhara region, encompassing the targeted study area.

## Methods

### Study design, period and setting

A prospective follow up study was conducted at Debre Markos comprehensive specialized hospital East Gojjam Zone, Amhara regional state, Northwest Ethiopia from July1 /2023 to February 30/2024. The hospital is found 299 km northwest of Addis Ababa, a capital city of Ethiopia and 268 km from Bahir Dar, a capital of Amhara regional state. Debre Markos comprehensive specialized hospital is the only referral hospital in Gojjam. It is the referral center of three zones (i.e., East Gojjam, West Gojjam, some part of Awi and some part of Gohatsion north Shewa). It gives a routine and comprehensive health service for an estimated population of more than 5 million found in the zone and nearby border areas. The obstetrics and gynecology unit is one of the largest and most well-known departments in the hospital. it is staffed with a team of midwives, obstetricians & gynecologists, and general practitioners. The department includes an Obstetrics and Gynecologic Emergency Unit, a Maternity Ward, a Labor and Delivery Room, and an Operating Room. Additionally, the hospital has a central Intensive Care Unit (ICU) that serves all patients, including obstetrics and gynecology cases. Annually, the hospital provides approximately 5,000 to 7,000 labor and delivery services. It also delivers an average of 908 ANC services, 2,347 PNC services, 5,298 prevention of mother-to-child transmission (PMTCT) of HIV/AIDS services, 2,347 comprehensive abortion care services, 1,035 cervical cancer screening services, and performs 2,347 cesarean sections (according to the hospital’s 2022 report). The armed conflict in Amhara escalated in August 2023 when Fano militants took control of several cities and towns (26). In response to the hostilities, the Ethiopian government declared a state of emergency in the Amhara region on August 4, 2023, which lasted for almost a year (27, 28). This situation disrupted transportation, particularly at night, significantly hindering access to obstetric care in emergency situations. Road closures further exacerbated the challenges of emergency transportation, while the blockage of the Ethio-telecom network added to the difficulty of coordinating emergency vehicles (29, 30). The conflict caused a severe human and resource crisis, particularly impacting the region’s healthcare system. The East Gojjam zone, where the hospital is located, experienced some of the most intense battles and continues to face the effects of the armed conflict.

### Populations

The study included all women who sought healthcare in the hospital during pregnancy, labor, delivery, or within 42 days after delivery or termination of pregnancy with in the study period.

### Eligibility criteria

All women who sought healthcare in the hospital during pregnancy, labor, delivery, or within 42 days after delivery or termination of pregnancy with in the study period were included without any restriction. Whereas, women presented with complication or seek treatment in the hospital after 42 days of delivery and women who died with accidental event were excluded from the study.

### Selection of the cases and sample size determination

All women who presented at the hospital during pregnancy, labor, delivery, or within 42 days after delivery or termination of pregnancy within the study period were included. From this cohort, women with potentially life-threatening conditions (PLTC), such as severe postpartum hemorrhage (PPH), pre-eclampsia with severity features, eclampsia, uterine rupture, severe complications of abortion or ectopic pregnancy, and sepsis/severe systemic infections, were identified based on WHO criteria (12). Subsequently, women with PLTC were selected, and among them, those with Maternal Near Miss (MNM) were identified using criteria specific to SSA and WHO guidelines (Supplementary Table S1). The identification of cases was made by the data collectors. And the confirmation of identified cases was carried out by the authors. All women who presented to study facility while pregnant, during child birth and/or within 42 days after termination of pregnancy seeking care and found to have PLTC and/ SMO during the study period were included as a sample.

### Variable of the study

Outcome: Severe maternal outcome (MNM and maternal death).

Independent: Sociodemographic variable (age, residence, referral status, distance from the hospital, mode of transportation), obstetrics related variable (gravidity, parity, previous mode of delivery, prenatal follow up, gestational age and place of delivery) and underlining medical problem.

### Measurement

Potentially life-threatening condition: A woman who had experienced at least one severe pregnancy-related complication, as classified by the World Health Organization (12).

Maternal near miss: Refers to a woman who nearly died but survived a complication that occurred during pregnancy, childbirth, or within 42 days of termination of pregnancy (12).

Maternal death (MD): The death of a woman while pregnant or within 42 days of termination of pregnancy from any cause related to or aggravated by the pregnancy or its management but not from accidental or incidental causes (31, 32).

Severe maternal outcome: Maternal near miss and maternal death (12).

Referred cases: Refers to women coming from health centers and district hospitals with existing complications.

Live birth (LB): The complete expulsion or extraction of a fetus from a woman, regardless of the duration of pregnancy, that shows any signs of life after separation (33).

Maternal mortality ratio (MMR): Is defined as the number of maternal deaths during a given time period per 100,000 live births during the same time period (34).

Mortality index (MI): The number of maternal deaths divided by the number of women who experienced severe maternal outcome (SMO), expressed as: [MI = MD/(MNM + MD)] (12).

Severe maternal outcome incidence ratio: The number of SMO (MNM&MD) cases per 1,000 live births; the numerator being the magnitude of SMO and the denominator is live births conducted at the hospitals during the specified period.

Maternal near-miss mortality ratio (MNM: MD): The ratio between maternal near miss and maternal deaths. Higher ratios show better care (12).

### Data collection tool and procedure

A structured and standard questionnaire was adapted from WHO and SSA near miss questionnaire. The question includes the socio-demographic, obstetrics related characteristics of the study participants. And near miss related questions includes: the clinical condition, laboratory investigations or laboratory-based criteria, and clinical intervention or management-based criteria. The data were collected by two BSC and two MSc midwives in the maternity ward, ICU, labor and delivery room, the obstetrics and gynecology emergency units every day during the study period; and they fill the required information of the patient until discharge based on the WHO and SSA near miss criteria. The patient charts, delivery and operation registration books were used as source of information to complete the questionnaire. In the case of detecting incomplete and or unclear information from the patients’ and/or facilities’ document; face-to-face interview with the women and/or their family (if she is dead) and the health care providers who managed her was employed to collect the required information. Information about referral status was also collected from their family, referral paper and health care providers in the hospital who received and examine the women. The data collection process was supervised and mentored by one obstetrician and gynecologist.

### Data quality control

The questionnaire was prepared in English. A pretest of the questionnaire was done on 5% of the participant (the sample size) at Finote-Selam general hospital in order to check the clarity, understandability and simplicity of the study tools. The data collectors and the supervisors were received training on how to collect the data correctly and appropriately and how to supervise the data collection process closely. The principal investigator, the 4th and the 5th authors were overseeing the entire data collection process and work closely with the supervisors.

### Data processing and analysis

The data were checked, coded and entered through Epi- data v. 4.6 and then export to the statistical package for social sciences (SPSS v.27) for analysis. Cross tabulation and descriptive statistics were carried out to clean the data, to check completeness of the data and to identify missing value and unusual or outlier data. Descriptive statistics of indicators of MNM and process indicators were calculated. SMO ratio, MNM ratio, MI and MMR were calculated. The results were presented in accordance with the WHO MNM approach, using clinical, laboratory and management criteria (12).

### Ethical consideration

Ethical clearance was obtained from Debre Berhan University Asrat Woldeyes health science campus Institutional Review Board (IRB) (Ref. NO: P0004/28/24). And formal letter of study approval was obtained from east Gojjam zonal health office and from Debre Markos specialized hospital. Finally written and oral consent was taken from each individual study participants and parents (if dead) after informed about the important of the study, how the Study carried out, the potential risks and benefits and all about the study. Participants address, names and signs could not be included in order to keep participants confidentiality.

## Results

**Figure 1:**
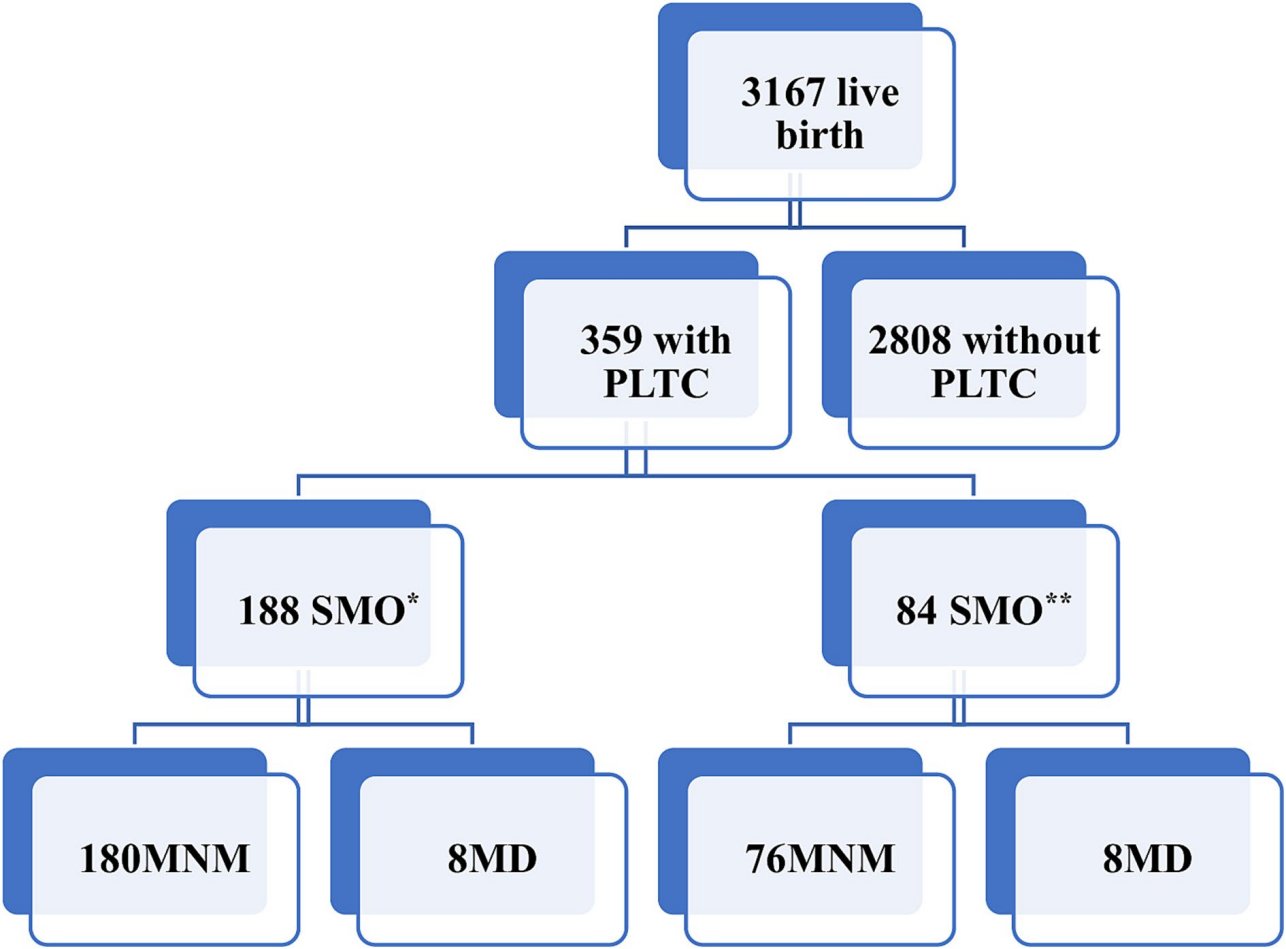
Study flow chart of severe maternal outcomes in east Gojjam. ^*^Based on sub-Saharan Africa MNM criteria, ^**^based on WHO MNM criteria, MD, maternal death.

During the study period, there were 3,167 live births in the study area. During this time, 359 women were admitted with PLTC. Of these cases, 188 were classified as SMO, which included 180 instances MNM and 8 maternal deaths based on SSA MNM criteria (Figure 1). Resulting in SMO ratio of 59.4 per 1,000 of live birth (95% CI: 51, 68 per 1,000 live birth). According to WHO criteria, the SMO cases numbered 84, with 76 MNM and 8 maternal deaths (26.5 per 1,000 liv births). The discrepancy in MNM cases between the SSA and WHO criteria is primarily due to variations in the recognition of Laparotomy other than cesarean section and the thresholds for blood transfusion. The threshold in the SSA is 2 units compared to five in the WHO criteria (Table 1).

**Table 1:** Distribution of SMO according to SSA and WHO criteria in east Gojjam, northwest Ethiopia, 2024 (*n* = 188).

Morbidity	SSA (*n*)	WHO (*n*)
Maternal near miss	180	76
Maternal death	8	8
Clinical criteria		
Acute cyanosis	3	3
Gasping	3	3
Respiratory rate > 40 or < 6 birth/min	15	15
Shock	49	49
Oliguria non responsive to fluid or diuretics	2	2
Failure to form clot	7	7
Loss of consciousness lasting ≥12 h	2	2
Cardiac arrest	4	4
Stroke	1	1
Uncontrollable fit/total paralysis or status epilepticus	0	0
Jaundice in the presence of preeclampsia	1	1
Eclampsia	30	8
Uterine rupture	35	15
Sepsis /severe systemic infection	9	3
Pulmonary edema	5	4
Severe complication of abortion^*^	14	5
Severe malaria	0	0
Laboratory based criteria		
Oxygen saturation < 90% for more than 60 min	11	11
Creatinine ≥3.5 mg/dL	2	2
Thrombocytopenia (<50,000 platelet/ml)	8	8
Loss of consciousness and keto acidosis in urine	4	4
Management based criteria		
Hysterectomy following hemorrhage/rupture or infection	25	25
Use of blood product >2 unit	97	31
Intubation and ventilation for >60 min unrelated to anesthesia	6	6
Cardiopulmonary resuscitation	4	4
Laparotomy other than cesarean section	76	37
Severe preeclampsia- eclampsia with ICU admission	10	10
Total^**^	422	259

* Severe complications of abortion include complicated ectopic pregnancies and abortion-related issues, such as incomplete abortion with significant bleeding that causes the woman in shock or massive transfusion.

** The total exceeds the number of SMO cases because some women meet more than one inclusion criterion.

### Characteristics of the study participants

The mean age of the study participants was 27.23 years, with a standard deviation of 5.23 years. Most of the participants (77.7%) were between the ages of 21 and 34. Most of women with SMO were residing from urban area (68.6%). Most women with SMO traveled to the hospital by taxi (Bajaj), accounting for 40.9% of the cases. The majority of participants had at least one antenatal care (ANC) visit during their current pregnancy (61.9%). A significant portion of women with SMO were multiparous (62.8%). Additionally, more than half of women with SMO were referred from other health facilities (55.9%) (Table 2).

**Table 2:** The sociodemographic and obstetrics characteristics of women with severe maternal outcomes in east Gojjam, northwest Ethiopia, 2024.

Variables		PLTC (*n* = 359)	SMO (*n* = 188)	Maternal death (*n* = 8)
Age	≤20 year	39 (10.9%)	24 (12.8%)	0
	21–34 year	279 (77.7%)	138 (73.9%)	3 (37.5%)
	35–49 year	41 (11.4%)	26 (13.8%)	5 (62.3%)
Marital status	Cohabited	53 (14.8%)	23 (12.2%)	1 (12.5%)
	Married	306 (85.2%)	165 (87.8%)	7 (87.5%)
Residence	Urban	246 (68.5%)	129 (68.6%)	4 (50%)
	Rural	113 (31.5%)	59 (31.4%)	4 (50%)
Educational status	No read & write	64 (17.8%)	32 (17%)	2 (25%)
	Read & write	38 (10.6%)	19 (10%)	3 (37.5%)
	Primary	78 (21.7%)	33 (17.6%)	1 (12.5%)
	Secondary	78 (21.7%)	46 (24.5%)	1 (12.5%)
	College & above	101 (28.1%)	58 (30.9%)	1 (12.5%)
Occupation	House wife	141 (39.3%)	74 (39.4%)	2 (25%)
	Gov’t employee	88 (24.5%)	53 (28.2%)	2 (25%)
	Student	16 (4.5%)	9 (4.8%)	0
	Private employee	34 (9.5%)	17 (9%)	1 (12.5%)
	Farmer	80 (22.3%)	35 (18.6%)	3 (37.5%)
Distance from hospital	>30 km from the hospital	167 (46.5%)	78 (41.5%)	6 (75%)
	<30 km from the hospital	192 (53.5%)	110 (58.5%)	2 (25%)
Mode of transportation to the hospital	Ambulance	61 (17.1%)	35 (18.6%)	2 (25%)
	Public transport	105 (29.2%)	56 (29.8%)	2 (25%)
	Taxi (Bajaj)	147 (40.9%)	79 (42%)	4 (57.1%)
	Bare foot	34 (9.5%)	10 (5.3%)	0
Gestational age	<28 week	76 (21.2%)	27 (15%)	0
	28–37 week	121 (33.7%)	67 (35.6)	1 (12.5%)
	>37 week	167 (46.5%)	92 (48.9%)	7 (87.5%)
Parity	0	130 (36.3%)	70 (37.2%)	0
	1–5	218 (60.7%)	111 (59.1%)	6 (75%)
	>5	11 (3%)	7 (3.7%)	2 (25%)
ANC (at least one)	Yes	221 (61.6%)	101 (53.7%)	3 (37.5%)
	No	138 (38.4%)	87 (46.3%)	5 (62.5%)
Referred from other facilities	Yes	127 (35.4%)	100 (53.2%)	6 (75%)
	No	232 (64.6%)	88 (46.8%)	2 (25%)

### Severe maternal outcome indicators

The SMO ratio was 59.4 per 1,000 live births according to SSA criteria. For every maternal death, there were 23 MNM cases, resulting in a mortality index (MI) of 4.2% and a near-miss to mortality ratio of 22.5:1, indicating better care. According to WHO criteria, the SMO ratio was lower at 26.5 per 1,000 live births, but the MI was higher at 9.5%, and the near-miss to mortality ratio was lower at 10.5:1. Notably, most women with SMO (80.3%) already had complications upon arrival or developed them within 12 h of admission to the hospital. More than half (55.9%) of the women with SMO were referred from other health facilities. Almost all maternal deaths (6 out of 8) occurred within 12 h of hospital admission, resulting in MI of 4. Two women died after 12 h of hospital admission with SMO. The proportion of SMO was almost three times higher among women referred from other health institutions compared to those who developed SMO within the hospital. Surprisingly, only 7.4% of women with SMO were admitted to the ICU; and among the total number of maternal deaths, 3 (37.5%) occurred in the ICU. The MMR in the study area were 252.6 per 100,000 of live births (Table 3).

**Table 3:** Sever maternal outcome indicators in east Gojjam based on SSA criteria, northwest Ethiopia, 2024.

Outcomes	SMO indicators
All live births in the population under surveillance (number)	3,167
Potentially life threating conditions (number)	359
Severe maternal outcomes (SMO) cases (number)	188
Maternal deaths (number)	8
Maternal near-miss cases (number)	180
Overall, near-miss indicators	
Severe maternal outcome ratio (per 1,000 live births)	59.4
Maternal near-miss ratio (per 1,000 live births)	56.8
Maternal near-miss mortality ratio (MNM:MD)	22.5:1
Mortality index (%)	4.2
Hospital access indicators	
SMO cases presenting with organ dysfunction or maternal death within 12 h of hospital stay (SM012) (number)	151
Proportion of SMO12 cases among all SMO cases (number)	80.3
Proportion of SMO12 cases coming from other health facilities (%)	55.9
Women who died on arrival or within 12 h of hospital stay (number)	6
SMO12 mortality index (%)	4
Intrahospital care	
Intrahospital SMO cases (number)	37
Intrahospital SMO ratio (per 1,000 live births)	11.7
Women who died after 12 h of hospital stay (number)^*^	2
Intrahospital mortality index (%)	5.4
Intensive care unit admission	
Total number of ICU admission (number)	14
Percentage of ICU admission among women with SMO (%)	7.4
Women who died at ICU (number)	3
Percentage of maternal deaths occurred within ICU admission (%)	37.5

*The two mortality cases presented with SMO upon arrival, but they died after 12 h of admission in the ICU, and thus were counted as intrahospital deaths. However, no deaths were reported among the intrahospital SMO cases.

### Underlining causes of SMO

Hypertensive disorders of pregnancy were the leading underlying cause of SMO, accounting for 46.1%, followed by obstetric hemorrhage at 32.2%. Most maternal deaths (75%) were due to obstetric hemorrhage (uterine rapture and PPH), resulting in a mortality index (MI) of 3.2%. Half of these maternal deaths specifically followed uterine rupture, with a MI of 2.1%. Anemia was the most associated or contributory condition of SMO (Table 4).

**Table 4:** Underlying causes of life-threatening conditions and severe maternal outcomes based on SSA criteria in east Gojjam, northwest Ethiopia, 2024.

Underlining cause and associated conditions	Women with PLTC (*n* = 359)		MNM cases (n = 180)		Maternal death		MI
	*n*	%	*n*	%	*n*	%	%
Underlining conditions							
Pregnancy with abortive outcomes	77	21.4%	27	15%	0	0	0
Abortion	36	8.1%	13	7.2%	0	0	0
Ectopic pregnancy	41	11.7%	14	7.8%	0	0	0
Obstetrics hemorrhage	82	22.8%	58	32.2%	6	75%	3.2
Placental previa	7	1.9%	3	1.7%	0	0	0
Placental abruption	10	2.9%	0	0	0	0	0
PPH	30	8.4%	25	13.9%	2	25%	1.1
Uterine rupture	35	9.7%	30	16.7%	4	50%	2.1
Hypertensive disorder of pregnancy	149	41.5%	83	46.1%	1	12.5%	0.5
Preeclampsia with severity feature	121	23.1%	53	29.4%	0	0	0
Eclampsia	30	8.4%	30	16.7%	1	12.5%	0.5
Sepsis/sever systemic infection^*^	51	14.2%	12	6.7%	1	12.5%	0.5
Contributory causes/associated conditions	105	29.2%					
Anemia	105	29.2%	98	54.4%	6	75%	
Previous C/S scar	4	(1.1%)	2	1.1%	0	0	
Critical intervention							
Blood transfusion^**^	97	27%	97	51.6%	6	75%	
Laparotomy other than CS	77	21.4%	47	26.1%	4	50%	
ICU admission	14	3.9%	14	7.8%	3	37.5%	

* The cases involved severe pregnancy infections (either septic abortion or postpartum/post operative infections). The deaths were specifically due to septic shock, occurring three days after admission to the ICU.

** Transfusion of two or more units of blood.

### Process and outcome indicators of SMO

Nearly all women (99.9%) received an uterotonic drug during the management of the third stage of labor, to help the prevention of PPH, with oxytocin being the most frequently used. All women with severe PPH were treated with some type of uterotonic drug, and only one woman (3.3%) with severe uncontrollable PPH, despite the use of various uterotonic drugs, required a hysterectomy. All women with preeclampsia with severity features and eclampsia were managed with anticonvulsant drugs, primarily magnesium sulfate. Almost all women (97.1%) with uterine rupture had a laparotomy within 3 hours of hospital admission. Uterine rupture was the leading cause of maternal mortality in the study area, resulting in 126 maternal deaths per 100,000 live births alone. Overall, this demonstrates that the hospital provides high-quality care to the catchment area (Table 5).

**Table 5:** Process and outcome indicators related to specific conditions among women with SMO based on SSA criteria in east Gojjam, northwest Ethiopia, 2024.

Indicators	Number	%
Prevention of PPH		
Target population: women giving birth in health-care facilities	3,167	-
Oxytocin use	3,163	99.87%
Use of any uterotonic (including oxytocin)	3,165	99.9%
Treatment of severe PPH		
Target population: women with severe PPH	30	-
Oxytocin use	30	100%
Ergometrine	17	56.7%
Misoprostol	27	90%
Tranexamic acid	11	36.7%
Any of the above uterotonics	85	>100%
Removal of retained products	7	23.3%
Uterine compression suture (blench)	5	16.7%
Hysterectomy	1	3.3%
Proportion of cases with SMO	25	83.3%
Mortality	2	6.7%
Anticonvulsants for eclampsia		
Target population: women with eclampsia & preeclampsia with severity features	151	
Magnesium sulfate (Mgso4)	138	91.4%
Another anticonvulsant^*^	13	8.6%
Any anticonvulsant	151	100%
Proportion of cases with SMO	84	55.6%
Mortality	1	0.7%
Prevention of cesarean section related infection		
Target population: women undergoing cesarean section	830	
Prophylactic antibiotic during cesarean section	828	99.8%
Treatment for sepsis		
Target population: women with sepsis	51	
Parenteral therapeutic antibiotics	51	100%
Proportion of cases with SMO	12	2.4%
Mortality	1	1.2%
Ruptured uterus		
Target population: women with ruptured uterus	35	
Laparotomy	35	100%
Laparotomy after 3 h of hospital stay	1	2.9%
Proportion of cases with SMO	34	97.1%
Mortality	4	11.4%

*Specifically, diazepam was primarily administered when MgSO4 was contraindicated.

## Discussion

This study revealed the incidence of SMO in East Gojjam over an eight-month period, coinciding with a time of severe armed conflict in the area. The incidence of SMO was 59.4 per 1,000 live births (95% CI: 51, 68 per 1,000 live births). The incidence of MNM was 56.8 per 1,000 live births (95% CI: 49, 65 per 1,000 live births), and the MMR was 252.6 per 100,000 live births (95% CI: 100, 400 per 100,000 live births). The leading causes of SMO (i.e., 78.3% of all SMO cases) were hypertensive disorders of pregnancy and obstetrical hemorrhage, including uterine rupture. Uterine rupture was responsible for half (50%) of the maternal deaths, followed by PPH, which accounted for 25%. Together, these conditions contributed to 75% of the MMR.

The incidence of SMO in this study aligns with findings from studies conducted at Jimma University Teaching Hospital in Ethiopia and at the National Referral Hospital in Somaliland, Hargeisa Group Hospital, which reported incidences of 59.2 per 1,000 live births and 61.3 per 1,000 live births, respectively (15, 19). However, it is higher than finding from study in south Ethiopia and India which is 37.5 and 19.6 per 1,000 live births, respectively (20, 35). The discrepancy might be attributed to differences in the population and study area. The primary reason for this discrepancy is that the current study was conducted in a region severely affected by armed conflict. This situation has significantly impacted transportation, particularly emergency transport, including ambulances. As a result, laboring women or pregnant women with PLTC faced numerous challenges in reaching the hospital, exemplifying the “third delay” in accessing care. These transportation difficulties likely contributed to the higher SMO ratio observed in this study.

In contrast, the incidence of SMO in this study is lower than the incidence reported in a study conducted in Harar town, East Ethiopia, which documented an incidence rate of 84 per 1,000 live births (36). This discrepancy can be attributed to several factors, primarily the difference in the study periods, which span a gap of 7 years. During this time, significant improvements have been made in maternal and child health services, including advancements in healthcare infrastructure, better access to quality prenatal and postnatal care, and more effective maternal mortality prevention strategies. These enhancements have contributed to a notable reduction in maternal mortality rates when comparing the data from 2016 to 2017 to the earlier study period. Consequently, the improved healthcare services and interventions over the past 7 years have likely played a crucial role in reducing the incidence of SMO observed in the current study (37).

The MNM ratio observed in this study, which is 56.8 per 1,000 live births, aligns with the findings from a study conducted at Jimma University Teaching Hospital in Ethiopia 50 per 1,000 live births. However, it is significantly higher than the MNM ratios reported in other regions such as southwest Ethiopia, Addis Ababa, southern Ethiopia, and India, where the ratios are 37.5, 8.1, 33.3, and 16.3 per 1,000 live births, respectively (35, 38–41). It is also significantly higher than the global average MNM ratio of 18.67 per 1,000 live births (42). This notable difference in MNM ratios can be attributed to several factors, primarily the differences in study populations and geographic areas. And the other reason might be, the hospital in the current study serves as a referral center for a broad region including east Gojjam, west Gojjam, parts of Awi, and the north Shoa zone. Consequently, it covers a larger and potentially more diverse population compared to the previous studies mentioned. This broader catchment area likely includes a higher number of complicated cases, contributing to the increased MNM ratio. Moreover, the timing of the study coincided with a period of severe armed conflict in the region. The instability and associated challenges likely exacerbated the incidence of maternal complications. The armed conflict could have disrupted access to healthcare services, increased stress and trauma, and led to poorer health outcomes overall, thereby contributing to the higher MNM ratio observed in this study.

The MNM ratio reported in this study, is lower than the ratios found in several other analyses. Specifically, it is lower than the ratio of 208 per 1,000 live births from a secondary analysis of a national dataset by Ethiopian public health institutes, the ratio of 140 per 1,000 live births from a systematic review in Ethiopia, and the ratio of 73.64 per 1,000 live births from a systematic review in Africa (21, 43, 44). This difference may be due to several factors. First, the current study was conducted in a single referral hospital, which might underestimate the true incidence of MNM cases. A single hospital may not capture all cases occurring in the broader population. Second, this study was conducted during a period of severe armed conflict in the area. The conflict led to the closure of many major roads, which restricted access to the referral hospital for many women living in remote areas. Under normal circumstances, these women might have been able to reach the hospital for care. The conflict-induced inaccessibility likely resulted in fewer maternal admissions to the hospital, thereby contributing to the lower observed incidence of MNM in this study.

The MMR in this study, which is comparable to the national mortality rate of 267 per 100,000 live births, aligns closely with findings from a study conducted in Harar town, eastern Ethiopia, which reported an MMR of 378 per 100,000 live births (5, 36, 37). It is also consistent with a prolonged follow-up study (2000–2019) conducted on 86 conflict-affected countries worldwide, which reported an MMR of 396 per 100,000 live births (22). However, the MMR in this study is lower than findings from other regions, including Jimma University Teaching Hospital in Ethiopia, which reported an MMR of 876.9 per 100,000 live births, southwest Ethiopia with 625 per 100,000 live births, and Hargeisa Group Hospital in Somaliland, which reported 462.4 per 100,000 live births (15, 19, 38). The lower MMR in the current study compared to some other regions may be due to differences in the populations served, geographical factors, and advancements in maternal health services over different study periods.

The MMR reported in this study is significantly lower than those reported in studies from the Tigray region during the armed conflict in northern Ethiopia, Afghanistan, and Yemen where the MMRs were 840, 639 and 1,855 per 100,000 live births, respectively (25, 45, 46). This disparity may be due to differences in the study methodologies and the context of each region. The Tigray study was community-based, while this study is institution-based, which could underestimate the MMR. During the armed conflict, barriers such as a lack of emergency transportation and disrupted telecommunication systems prevented pregnant women from accessing obstetric care at hospitals or even primary healthcare facilities. Consequently, many women likely experienced severe life-threatening complications (MNM) or died at home before reaching medical facilities. The community-based nature of the Tigray study, covering a wider geographical area, may explain the higher MMR reported there. In Afghanistan and Yemen, the prolonged nature of the conflict likely played a significant role in the high MMR. Continuous and extended conflict severely disrupts health systems, depletes resources, and undermines the delivery of maternal healthcare services, and limits access to emergency obstetric care, and exacerbates risks for pregnant women. Contributing to a higher MMR in the region (47–49).

The MI in the current study is 4.2 which is comparable to the finding from a study in Harar, eastern Ethiopia, where the MI was 2.2 (36). However, the MI in this study is lower than the findings from Jimma University Teaching Hospital in Ethiopia (14.8), southwest Ethiopia (16.7), and Hargeisa Group Hospital in Somaliland (7.6) (15, 19, 20). Several factors may explain the differences in MI between the current study and others. Variations in study populations, geographic differences, and the periods during which the studies were conducted all contribute to differing MIs. Populations with higher risk factors or limited access to healthcare, and regions with challenging terrains or less developed infrastructure, may experience higher MIs. Improvements in healthcare infrastructure, availability of skilled providers, and maternal care programs over time can lead to better outcomes. The lower MI in the current study indicates better quality of maternal care in the study hospital.

The leading causes of SMO in this study are HDP followed by obstetrical hemorrhage. These findings are consistent with studies conducted in south Ethiopia, Harar in eastern Ethiopia, Tigray during the time of northern Ethiopia conflict, and Jimma University Teaching Hospital in Ethiopia (19, 20, 25, 36). However, a study conducted at Hargeisa Group Hospital in Somaliland reported that the leading cause of SMO is obstetric hemorrhage, followed by HDP (15). This difference may be attributed to variations in study populations and geographical areas. Different regions and populations may experience varying prevalences of maternal health issues due to factors such as genetic predispositions, availability and quality of healthcare services, socioeconomic conditions, and cultural practices.

In this study, uterine rupture was identified as the leading cause of maternal death (50%), followed by obstetrical hemorrhage (25%), eclampsia (12.5%), and sepsis (12.5%). This contrasts with national reports where obstetrical hemorrhage was predominant (40%), followed by hypertensive disorders of pregnancy (HDP) (17.6%), and uterine rupture (10%). In Harar, eastern Ethiopia, HDP accounted for the majority (50%) of maternal deaths, with obstetrical hemorrhage following closely (42.9%), and uterine rupture contributing 7.1%. Similarly, in south Ethiopia, HDP was the leading cause (61.1%), followed by obstetrical hemorrhage (27.8%), and uterine rupture (11.1%). At Jimma University Teaching Hospital in Ethiopia, HDP accounted for 37.5% of maternal deaths, with obstetrical hemorrhage at 29%, and uterine rupture at 8.3%. These findings are consistent with similar patterns observed in Somaliland (15, 19, 20, 36, 50). The primary difference in this study may be attributed to the severe armed conflict occurring during the study period. During this time, transportation, including ambulance services and public transit, was disrupted or unavailable. As a result, laboring women from remote areas or those referred from distant health facilities faced significant challenges in reaching the hospital. These transportation disruptions contributed to what is known as the third delay in obstetric care, where delays in accessing medical services can lead to complications. In this context, women experiencing prolonged labor due to delays in reaching medical care may develop obstructed labor, which can ultimately result in uterine rupture upon presentation at the hospital. This sequence of events underscores why uterine rupture emerged as the leading cause of maternal death in this study. The disruptions in transportation and delays in accessing timely medical interventions exacerbated by the armed conflict highlight the critical importance of ensuring uninterrupted access to obstetric care services, particularly in conflict-affected regions, to prevent such tragic outcomes.

### Limitation of the study

The limitations of this study include its single-institution setting and the period during severe armed conflict in the area, which may potentially lead to an underestimation or overestimation of the incidence of SMO and its contributing causes within the region. Conducting studies in a single institution and an unstable setting may not capture the full spectrum of maternal health issues across broader populations with varying healthcare access and socio-economic backgrounds. Consequently, generalizing the findings from this study to the wider population should be approached with caution. The diversity of healthcare settings and patient demographics in the general population could present different patterns of SMO that were not fully represented in this study’s hospital-based sample. Future research incorporating multiple healthcare institutions and diverse patient populations would provide a more comprehensive understanding of maternal health challenges and outcomes in the region.

## Conclusion

This study found that the incidence of SMO was comparable to that reported in most other studies. HDP emerged as the primary cause of SMO, followed by obstetrical hemorrhage, which aligns with similar studies conducted in Ethiopia. Notably, uterine rupture was identified as the leading cause of maternal mortality during the study period in the study area. The high incidence of SMO highlights the urgent need for targeted interventions and improved access to comprehensive maternal healthcare services. Addressing these leading causes through effective healthcare strategies, including early detection, timely intervention, and improved obstetric care, is essential for reducing maternal mortality and improving maternal health outcomes in similar settings.

## Data Availability

The raw data supporting the conclusions of this article will be made available by the authors, without undue reservation.
