# Supplementary material for: Incidence of severe maternal outcomes following armed conflict in East Gojjam zone, Amhara region, Ethiopia: using the sub-Saharan Africa maternal near-miss criteria
Source: Front Public Health. 2025 Jan 8;12:1456841. doi: 10.3389/fpubh.2024.1456841 (PMC11751003; doi:10.3389/fpubh.2024.1456841)
Supplement: Supplementary file 1 [file Table_1.DOCX]

S1: Sub-Saharan and WHO maternal near miss criteria tool

| WHO criteria | Sub-Saharan Africa criteria |
| --- | --- |
| Clinical criteria |  |
| Acute cyanosis | Acute cyanosis |
| Gasping | Gasping |
| Respiratory rate >40 or <6/minute | Respiratory rate >40 or <6/minute |
| Oliguria nonresponsive to fluids or diuretics | Oliguria nonresponsive to fluids or diuretics |
| Shock | Shock |
| Failure to form clots | Failure to form clots |
| Loss of consciousness lasting >/=12hr | Loss of consciousness lasting >/=12hr |
| Cardiac arrest | Cardiac arrest |
| Strock | Strock |
| Uncontrollable fit/total paralysis | Uncontrollable fit/total paralysis |
| Jaundice in the presence of preeclampsia | Jaundice in the presence of preeclampsia |
|  | Eclampsia |
|  | Uterine rupture |
|  | Sepsis or severe systemic infection |
|  | Pulmonary edema |
|  | Severe abortion complications |
|  | Severe malaria |
|  | Severe pre-eclampsia with ICU admission |
| Laboratory based criteria |  |
| Oxygen saturation<90%for >60 minute | Oxygen saturation<90%for >60 minute |
| Creatinine >300μmol/l or >3.5 mg/dl | Creatinine >300μmol/l or >3.5 mg/dl |
| PaO2/FiO2<200mmhg |  |
| Bilirubin >100 μmol/l or >6.0 mg/dl |  |
| Lactate >5 mEq/ml |  |
| pH <7.1 |  |
| Acute thrombocytopenia (<50,000 platelets/ml) | Acute thrombocytopenia (<50,000 platelets/ml) |
| Loss of consciousness and ketoacids in urine | Loss of consciousness and ketoacids in urine |
| Management based criteria |  |
| Use of continuous vasoactive drugs |  |
| hysterectomy following infection or hemorrhage | hysterectomy following infection or hemorrhage |
| Transfusion of >/=5 units of red blood cells | Transfusion of >/=2 units of red blood cells |
| Intubation and ventilation for >/=60 min not related to anesthesia | Intubation and ventilation for >/=60 min not related to anesthesia |
| Cardio-pulmonary resuscitation | Cardio-pulmonary resuscitation |
| Dialysis for acute renal failure |  |
