# Supplementary material for: Incidence of severe maternal outcomes following armed conflict in East Gojjam zone, Amhara region, Ethiopia: using the sub-Saharan Africa maternal near-miss criteria
Source: Front Public Health. 2025 Jan 8;12:1456841. doi: 10.3389/fpubh.2024.1456841 (PMC11751003; doi:10.3389/fpubh.2024.1456841)
Supplement: Supplementary file 2 [file Table_2.DOCX]

| Morbidity | SSA (n) | WHO(n) |
| --- | --- | --- |
| Maternal near miss | 180 | 76 |
| Maternal death | 8 | 8 |
| Clinical criteria |  |  |
| Acute cyanosis | 3 | 3 |
| Gasping | 3 | 3 |
| Respiratory rate >40 or <6 birth/minute | 15 | 15 |
| Shock | 49 | 49 |
| Oliguria non responsive to fluid or diuretics | 2 | 2 |
| Failure to form clot | 7 | 7 |
| Loss of consciousness lasting ≥12 hours | 2 | 2 |
| Cardiac arrest | 4 | 4 |
| Stroke | 1 | 1 |
| Uncontrollable fit/total paralysis or status epilepticus | 0 | 0 |
| Jaundice in the presence of preeclampsia | 1 | 1 |
| Eclampsia | 30 | 8 |
| Uterine rupture | 35 | 15 |
| Sepsis /severe systemic infection | 9 | 3 |
| Pulmonary edema | 5 | 4 |
| Severe complication of abortion^*^ | 14 | 5 |
| Severe malaria | 0 | 0 |
| Laboratory based criteria |  |  |
| Oxygen saturation <90% for more than 60 minute | 11 | 11 |
| Creatinine ≥3.5mg/dl | 2 | 2 |
| Thrombocytopenia (<50,000 platelet/ml) | 8 | 8 |
| Loss of consciousness and keto acidosis in urine | 4 | 4 |
| Management based criteria |  |  |
| Hysterectomy following hemorrhage/rupture or infection | 25 | 25 |
| Use of blood product > 2 unit | 97 | 31 |
| Intubation and ventilation for >60minute unrelated to anesthesia | 6 | 6 |
| Cardiopulmonary resuscitation | 4 | 4 |
| Laparotomy other than caesarean section | 76 | 37 |
| Severe preeclampsia- eclampsia with ICU admission | 10 | 10 |
| Total^**^ | **422** | **259** |
| ^*^ Severe complications of abortion include complicated ectopic pregnancies and abortion-related issues, such as incomplete abortion with significant bleeding that causes the woman in shock or massive transfusion.  ^**^ The total exceeds the number of SMO cases because some women meet more than one inclusion criterion. | | |
