# Supplementary material for: Incidence of severe maternal outcomes following armed conflict in East Gojjam zone, Amhara region, Ethiopia: using the sub-Saharan Africa maternal near-miss criteria
Source: Front Public Health. 2025 Jan 8;12:1456841. doi: 10.3389/fpubh.2024.1456841 (PMC11751003; doi:10.3389/fpubh.2024.1456841)
Supplement: Supplementary file 3 [file Table_3.DOCX]

| Variables | | PLTC (n=359) | SMO (n=188) | Maternal death (n=8) |
| --- | --- | --- | --- | --- |
| Age | ≤20 year | 39(10.9%) | 24 (12.8%) | 0 |
|  | 21-34 year | 279(77.7%) | 138(73.9%) | 3(37.5%) |
|  | 35-49 year | 41(11.4%) | 26(13.8%) | 5(62.3%) |
| Marital status | Cohabited | 53 (14.8%) | 23(12.2%) | 1(12.5%) |
|  | Married | 306(85.2%) | 165(87.8%) | 7(87.5%%) |
| Residence | Urban | 246(68.5%) | 129(68.6%) | 4(50%) |
|  | Rural | 113(31.5%) | 59(31.4%) | 4(50%) |
| Educational status | No read & write | 64(17.8%) | 32(17%) | 2(25%) |
|  | Read & write | 38(10.6%) | 19(10%) | 3(37.5%) |
|  | Primary | 78(21.7%) | 33(17.6%) | 1(12.5%) |
|  | Secondary | 78(21.7%) | 46(24.5%) | 1(12.5%) |
|  | College & above | 101(28.1%) | 58(30.9%) | 1(12.5%) |
| Occupation | House wife | 141(39.3%) | 74(39.4%) | 2(25%) |
|  | Gov`t employee | 88(24.5%) | 53(28.2%) | 2(25%) |
|  | Student | 16(4.5%) | 9(4.8%) | 0 |
|  | Private employee | 34(9.5%) | 17(9%) | 1(12.5%) |
|  | Farmer | 80(22.3%) | 35(18.6%) | 3(37.5%) |
| Distance from hospital | >30km from the hospital | 167(46.5%) | 78(41.5%) | 6(75%) |
|  | <30km from the hospital | 192(53.5%) | 110(58.5%) | 2(25%) |
| Mode of transportation to the hospital | Ambulance | 61(17.1%) | 35(18.6%) | 2(25%) |
|  | Public transport | 105(29.2%) | 56(29.8%) | 2(25%) |
|  | Taxi (Bajaj) | 147(40.9%) | 79(42%%) | 4(57.1%) |
|  | Bare foot | 34(9.5%) | 10(5.3%) | 0 |
| Gestational age | <28 week | 76 (21.2%) | 27(15%) | 0 |
|  | 28- 37 week | 121(33.7%) | 67(35.6) | 1(12.5%) |
|  | >37 week | 167(46.5%) | 92(48.9%) | 7 (87.5%) |
| Parity | 0 | 130(36.3%) | 70(37.2%) | 0 |
|  | 1-5 | 218(60.7%) | 111(59.1%) | 6(75%) |
|  | >5 | 11(3%) | 7(3.7%) | 2(25%) |
| ANC (at least one) | Yes | 221(61.6%) | 101(53.7%) | 3(37.5%) |
|  | No | 138(38.4%) | 87(46.3%) | 5(62.5%) |
| Referred from other facilities | Yes | 127(35.4%) | 100(53.2%) | 6(75%) |
|  | No | 232(64.6%) | 88(46.8%) | 2(25%) |
