# Supplementary material for: Incidence of severe maternal outcomes following armed conflict in East Gojjam zone, Amhara region, Ethiopia: using the sub-Saharan Africa maternal near-miss criteria
Source: Front Public Health. 2025 Jan 8;12:1456841. doi: 10.3389/fpubh.2024.1456841 (PMC11751003; doi:10.3389/fpubh.2024.1456841)
Supplement: Supplementary file 4 [file Table_4.DOCX]

| Outcomes | SMO indicators |
| --- | --- |
| All live births in the population under surveillance (number) | 3167 |
| Potentially life threating conditions (number) | 359 |
| Severe maternal outcomes (SMO) cases (number) | 188 |
| Maternal deaths (number) | 8 |
| Maternal near-miss cases (number) | 180 |
| Overall, near-miss indicators |  |
| Severe maternal outcome ratio (per 1000 live births) | 59.4 |
| Maternal near-miss ratio (per 1000 live births) | 56.8 |
| Maternal near-miss mortality ratio (MNM:MD) | 22.5:1 |
| Mortality index (%) | 4.2 |
| Hospital access indicators |  |
| SMO cases presenting with organ dysfunction or maternal death within 12 hours of hospital stay (SM012) (number) | 151 |
| Proportion of SMO12 cases among all SMO cases (number) | 80.3 |
| Proportion of SMO12 cases coming from other health facilities (%) | 55.9 |
| Women who died on arrival or within 12hrs of hospital stay(number) | 6 |
| SMO12 mortality index (%) | 4 |
| Intrahospital care |  |
| Intrahospital SMO cases (number) | 37 |
| Intrahospital SMO ratio (per 1000 live births) | 11.7 |
| Women who died after 12hrs of hospital stay(number)^*^ | 2 |
| Intrahospital mortality index (%) | 5.4 |
| Intensive care unit admission |  |
| Total number of ICU admission(number) | 14 |
| Percentage of ICU admission among women with SMO (%) | 7.4 |
| Women who died at ICU (number) | 3 |
| Percentage of maternal deaths occurred within ICU admission (%) | 37.5 |
| ^*^The two mortality cases presented with SMO upon arrival, but they died after 12 hours of admission in the ICU, and thus were counted as intrahospital deaths. However, no deaths were reported among the intrahospital SMO cases. | |
