# Supplementary material for: Incidence of severe maternal outcomes following armed conflict in East Gojjam zone, Amhara region, Ethiopia: using the sub-Saharan Africa maternal near-miss criteria
Source: Front Public Health. 2025 Jan 8;12:1456841. doi: 10.3389/fpubh.2024.1456841 (PMC11751003; doi:10.3389/fpubh.2024.1456841)
Supplement: Supplementary file 5 [file Table_5.DOCX]

| Underlining cause and associated conditions | Women with PLTC (n=359) | | MNM cases (n=180) | | Maternal death | | MI |
| --- | --- | --- | --- | --- | --- | --- | --- |
|  | n | % | n | % | n | % | % |
| Underlining conditions |  |  |  |  |  |  |  |
| Pregnancy with abortive outcomes | **77** | **21.4%** | **27** | **15%** | **0** | **0** | **0** |
| Abortion | 36 | 8.1% | 13 | 7.2% | 0 | 0 | 0 |
| Ectopic pregnancy | 41 | 11.7% | 14 | 7.8% | 0 | 0 | 0 |
| Obstetrics hemorrhage | **82** | **22.8%** | **58** | **32.2%** | **6** | **75%** | **3.2** |
| Placental previa | 7 | 1.9% | 3 | 1.7% | 0 | 0 | 0 |
| Placental abruption | 10 | 2.9% | 0 | 0 | 0 | 0 | 0 |
| PPH | 30 | 8.4% | 25 | 13.9% | 2 | 25% | 1.1 |
| Uterine rupture | 35 | 9.7% | 30 | 16.7% | 4 | 50% | 2.1 |
| Hypertensive disorder of pregnancy | **149** | **41.5%** | **83** | **46.1%** | **1** | **12.5%** | **0.5** |
| Preeclampsia with severity feature | 121 | 23.1% | 53 | 29.4% | 0 | 0 | 0 |
| Eclampsia | 30 | 8.4% | 30 | 16.7% | 1 | 12.5% | 0.5 |
| Sepsis/sever systemic infection^*^ | **51** | **14.2%** | **12** | **6.7%** | **1** | **12.5%** | **0.5** |
| Contributory causes/associated conditions | 105 | 29.2% |  |  |  |  |  |
| Anemia | 105 | 29.2% | 98 | 54.4% | 6 | 75% |  |
| Previous C/S scar | 4 | (1.1%) | 2 | 1.1% | 0 | 0 |  |
| Critical intervention |  |  |  |  |  |  |  |
| Blood transfusion^**^ | 97 | 27% | 97 | 51.6% | 6 | 75% |  |
| Laparotomy other than CS | 77 | 21.4% | 47 | 26.1% | 4 | 50% |  |
| ICU admission | 14 | 3.9% | 14 | 7.8% | 3 | 37.5% |  |
| ^*^ The cases involved severe pregnancy infections (either septic abortion or postpartum/post operative infections). The deaths were specifically due to septic shock, occurring three days after admission to the ICU.  ^**^ Transfusion of two or more units of blood | | | | | | |  |
