# Supplementary material for: Incidence of severe maternal outcomes following armed conflict in East Gojjam zone, Amhara region, Ethiopia: using the sub-Saharan Africa maternal near-miss criteria
Source: Front Public Health. 2025 Jan 8;12:1456841. doi: 10.3389/fpubh.2024.1456841 (PMC11751003; doi:10.3389/fpubh.2024.1456841)
Supplement: Supplementary file 6 [file Table_6.DOCX]

| Indicators | Number | % |
| --- | --- | --- |
| Prevention of PPH |  |  |
| Target population: women giving birth in health-care facilities | 3,167 | - |
| Oxytocin use | 3,163 | 99.87% |
| Use of any uterotonic (including oxytocin) | 3,165 | 99.9% |
| Treatment of severe PPH |  |  |
| Target population: women with severe PPH | 30 | - |
| Oxytocin use | 30 | 100% |
| Ergometrine | 17 | 56.7% |
| Misoprostol | 27 | 90% |
| Tranexamic acid | 11 | 36.7% |
| Any of the above uterotonics | 85 | >100% |
| Removal of retained products | 7 | 23.3% |
| Uterine compression suture (blench) | 5 | 16.7% |
| Hysterectomy | 1 | 3.3% |
| Proportion of cases with SMO | 25 | 83.3% |
| Mortality | 2 | 6.7% |
| Anticonvulsants for eclampsia |  |  |
| Target population: women with eclampsia & preeclampsia with severity features | 151 |  |
| Magnesium sulfate (Mgso4) | 138 | 91.4% |
| Another anticonvulsant^*^ | 13 | 8.6% |
| Any anticonvulsant | 151 | 100% |
| Proportion of cases with SMO | 84 | 55.6% |
| Mortality | 1 | 0.7% |
| Prevention of caesarean section related infection |  |  |
| Target population: women undergoing caesarean section | 830 |  |
| Prophylactic antibiotic during caesarean section | 828 | 99.8% |
| Treatment for sepsis |  |  |
| Target population: women with sepsis | 51 |  |
| Parenteral therapeutic antibiotics | 51 | 100% |
| Proportion of cases with SMO | 12 | 2.4% |
| Mortality | 1 | 1.2% |
| Ruptured uterus |  |  |
| Target population: women with ruptured uterus | 35 |  |
| Laparotomy | 35 | 100% |
| Laparotomy after 3 hours of hospital stay | 1 | 2.9% |
| Proportion of cases with SMO | 34 | 97.1% |
| Mortality | 4 | 11.4% |
| *Specifically, diazepam was primarily administered when MgSO4 was contraindicated. | | |
